# Comparison standards shape everyday judgments of low and high wellbeing in individuals with and without psychopathology: a diary-based investigation

**DOI:** 10.1038/s41598-024-54681-x

**Published:** 2024-02-19

**Authors:** Thomas Meyer, Marthe Sickinghe, Vanessa Matera, Nexhmedin Morina

**Affiliations:** Institute of Psychology, University of Münster, Münster, Germany

**Keywords:** Psychology, Quality of life, Psychology and behaviour, Psychiatric disorders

## Abstract

People can easily rate and express their current levels of wellbeing, but the cognitive foundations for such judgments are poorly understood. We examined whether comparisons to varying standards underlie fluctuating wellbeing judgments within-person (i.e., throughout daily episodes) and between-person (i.e., high vs. low levels of psychopathology). Clinical and non-clinical participants recorded subjective affect for each distinct episode for one week. Participants briefly described current, best, and worst daily episodes, which we coded for presence and type of comparison standard (social, past temporal, criteria-based, counterfactual, prospective temporal, and dimensional). Participants also rated their engagement with these standards and the respective affective impact. During best episodes, participants reported more downward (vs. upward) comparisons that resulted in positive affective impact. In worst episodes, upward (vs. downward) comparisons were more frequent. In best and worst episodes, we most frequently identified past-temporal and criteria-based comparisons, respectively. The clinical group engaged more often with all potential standard types during worst daily episodes and was more negatively affected by comparative thoughts, amid consistently more negative affect levels across all episode types. Our data suggest that judgments of affect and wellbeing may indeed rely on comparative thinking, whereby certain standards may characterize states of negative affect and poor mental health.

## Introduction

People constantly evaluate aspects of their own life and can easily indicate how they are doing in virtually any domain, including their emotional, psychological, physical, economic, or social wellbeing. The ability to form such judgments—broadly summarized under the term *subjective wellbeing*^1^—is the basis for self-awareness and effective regulation of cognition, emotion, and behavior. Despite some proneness to error and susceptibility to various biases^2–4^, simple ratings of wellbeing can serve as reliable predictors of psychological and behavioral outcomes^5,6^, including mental health trajectories and symptoms of psychopathology^7–9^. Yet, surprisingly little is known about the cognitive mechanisms that underlie and shape subjective wellbeing. Here, we propose that comparative thinking plays a central role in subjective wellbeing and that comparisons can help us understand how subjective affect judgments can vary within and between individuals (e.g., good vs. bad episodes, in individuals with vs. without psychopathology).

Frame-of-reference theories posit that all self-judgments are formed in relation to some point of reference^10–13^, rather than relying on fixed or absolute internal utility scales. For instance, decision-by-sampling theory^14^ postulates that mentally computing any value (e.g., to represent one’s current affect level on a 0–10 scale) requires ordinal comparisons against a series of internal reference points—or *comparison standards*—retrieved from memory or constructed through mental simulation. Thus, computation of a value for subjective affect (e.g., “How am I feeling?) is directly shaped by a set of standards (e.g., “better than yesterday”, “worse than my colleague”). In addition, subjective affect can also be influenced indirectly by comparisons on other self-relevant dimensions^11^. For example, comparisons on social media may carry motivational significance and influence affect (e.g., “When I see others showcasing their perfect bodies and luxurious vacations, it seems like I’m doing worse than everyone else, which makes me feel bad about myself”), even if the comparison dimensions per se (e.g., appearance, luxury) are only remote aspects of wellbeing. Moreover, the specificity of comparison standards may differ. An example for a specific social standard with respect to “being a good father” would be “my father as a parent”, whereas an example of a general social standard would be “thinking of how fathers should behave”. From these considerations it follows that subjective affect fluctuations are accompanied by some change in the comparison(s) that directly or indirectly shape affect. Similarly, individuals reporting chronically low levels of wellbeing (e.g., individuals with a mental disorder^5–9,15^) would be expected to rely on different comparisons (e.g., against relevant upward standards to which they perceive a larger discrepancy) than individuals with systematically higher affect levels (e.g., healthy individuals)—even if the pathways contributing to low levels of wellbeing may be extremely heterogenous.

To date, there is a scarcity of systematic research into comparison standards underlying fluctuations in subjective affect, in part due to lack of comprehensive framework of comparison types^11^. Yet, several standards informing self-perception are well-established, most prominently social (e.g. comparison to another person)^16,17^, but also temporal (e.g., comparison to prior experiences)^18^, criteria-based (e.g., comparison to an ideal version of the self)^19^, counterfactual (e.g., comparison to what could have been)^20^, or dimensional (e.g., comparing one's wellbeing to another life domain such as sports performance)^21^ standards. In principle, they can all serve to inform self-evaluations by indicating whether the standard is perceived as ranking higher (i.e., upward comparison), lower (i.e., downward comparison), or similarly (i.e., lateral comparison) on some self-attribute. A recurrent finding in the literature is that people feel worse following upward comparisons and better following downward comparison^17,22^. Hence, more upward comparisons would be expected in episodes with more negative subjective affect, whereas less negative episodes would be characterized by downward comparisons. Indeed, in a diary study, Wheeler and Miyake^23^ demonstrated that good and bad moods predicted reports of downward and upward comparisons, respectively. A similar case can be made for individuals with more positive vs. more negative affective states, such that people with chronically elevated negative affect can be expected to generally engage with different comparison standards than individuals with higher affect levels.

These important ideas await empirical investigation. For instance, despite potential affective costs, most people have been found to generally select upward social standards^17^, and experimental studies on comparison selection indicate that this is also the case in populations with elevated psychological distress, such as cardiac patients^24^ or dysphoric individuals^25^. Yet, there is growing evidence for meaningful differences in comparison standards between individuals, self-dimensions, and situations. For example, cross-sectional surveys into the role of comparisons in self-assessment of appearance^22,26^, academic abilities^27–29^, coping following a traumatic event^30^, or general wellbeing^31^ all show that self-perception is associated with multiple-standard comparisons, with domain-specific differences in the relative importance of the various standard types. A consistent finding across these studies is that the frequency of aversive comparisons (i.e., mostly upward) is associated with more negative psychological outcomes, such as appearance concerns, burnout, anxiety, or depression. In addition, studies into lifespan effects on subjective wellbeing have found robust age differences in the use of comparison standards^32–35^. For instance, in an online study with 2000 respondents, Filus et al.^36^ found that younger adults reported more engagement with interpersonal (i.e., social), imaginary (e.g., counterfactual), and downward standards than middle-aged and older participants. Together, these data make a strong case for the involvement of comparative thinking in self-judgments and mental health and point to a robust link between the frequency of upward comparisons and psychological distress. Moreover, there are indications that unfavorable social, criteria-based, past temporal, and counterfactual comparisons have a stronger negative influence on affect in clinically ill populations^20,37^.

However, the extant literature offers only a very limited insight into the dynamics of *everyday* comparative thinking that may underly fluctuations in subjective affect as well as differences between clinical and non-clinical populations. While a few studies have addressed naturally occurring comparisons^23^, these have not systematically measured affect dynamics in healthy and mentally ill populations using a broad framework of comparison standards. Yet, in theory, mental disorder might be characterized by aberrant comparisons in a variety of ways. For instance, treatment-seeking individuals with a mental disorder have lower levels of wellbeing^7–9^, making it likely that wellbeing is a relatively more salient personal attribute for them compared to non-clinical populations. Consequently, it can be expected that treatment-seeking individuals will generally be more likely to engage in wellbeing-related comparisons^37,38^. In addition, cognitive models of psychopathology posit the presence of information processing biases favoring negative information about the self^39,40^, which may lead to more frequent engagement in upward comparison, as well as a stronger negative affective impact of upward comparisons. Alternatively or in addition, weaker belief updating in response to positive information^40^ may be associated with less frequent downward comparison, combined with less positive affect derived from such “appetitive” comparisons.

To address this gap, the current study aimed to investigate more systematically how comparison standards vary within individuals (i.e., best vs. worst daily episodes) and between individuals (i.e., with vs. without psychopathology) in terms of comparison direction, type, and affective impact. We therefore invited treatment-seeking individuals diagnosed with mental disorders (clinical group), as well as matched individuals without a mental disorder (non-clinical group), to a 1-week diary study. All participants had previously participated in an interview study^41^, in which they were asked to substantiate ratings of current wellbeing. In the current study, they recorded affect ratings for each distinct episode once per day, by means of the Day Reconstruction Method^42^. Following each day reconstruction, they gave a brief explanation about their best and worst daily episode and rated the frequency and affective impact of thoughts about different comparison standards (social, past-temporal, criteria-based, counterfactual, prospective temporal, dimensional). We then coded the open-ended answers for the presence and type of comparison.

With respect to within-person differences, we expected that episodes with relatively negative affect (i.e., episodes judged to be the worst daily episodes) would be characterized generally by more upward than downward comparisons, relative to episodes with relatively positive affect (i.e., episodes judged to be the best daily episodes). We further expected the clinical group to display more upward comparisons across episodes, relative to the non-clinical group. Moreover, we expected the clinical group to display relatively larger negative impact of comparisons on mood, i.e.: a more negative impact of comparisons during the worst episodes, and a less positive impact during best episodes. Due to a scarcity of research, we did not initially formulate firm predictions concerning group differences per type of comparison or type of episode. Based on prior studies with clinical populations^20,37,41^, we tentatively expected the clinical group to display more upward social, criteria-based, past-temporal, and counterfactual comparisons, as well as a stronger negative influence on affect in clinically ill populations. Also on an exploratory basis, we investigated whether the association between self-reported comparison engagement and affective impact is more negative in the clinical than in the non-clinical group, extending our general hypothesis that comparisons are more detrimental for the clinical group. Finally, we explored whether the clinical group would engage in comparison with less concrete comparison standards.

## Method

### Participants

The current study was completed by 25 individuals seeking psychotherapy in the outpatient clinic for psychotherapy at the University of Münster, as well as 25 healthy individuals without clinical symptomatology matched on age, sex, and level of education. All participants enrolled after having completed a separate study, in which we used a semi-structured interview to assess comparative thinking^41^. For three additional participants from the interview study who completed this study, diary data were lost (technical failure: *n* = 1; filled out less than 15% of measurements: *n* = 2). Hence, these participants and their non-clinical counterparts were not included in any of the analyses. Inclusion criteria for the clinical group were proficiency in German at an intermediate level or higher and diagnosis of one or more mental disorders established with the Structured Clinical Interview for DSM-IV (SCID-I)^43^. Exclusion criteria were acute suicide risk, self-injury, substance addiction, a body mass index (BMI) < 17.5, acute psychotic symptoms (to exclude altered states of consciousness or severe formal thought disorders that may complicate open answer coding, e.g., due to loose or derailed associations, neologisms, stilted language, etc.), and/or current psychiatric or psychological treatment. For the nonclinical group, diagnosis or treatment of a mental disorder served as an additional exclusion criterion, established by means of a self-report screening, corroborated by below-cutoff depression and anxiety scores (see below; for details of the recruitment process, see Morina et al.^41^). Characteristics of the final sample are summarized in Table 1. Main diagnoses in the clinical group were mood (*n* = 8) and anxiety disorders (*n* = 8), adjustment disorder (*n* = 3), obsessive–compulsive disorder (*n* = 2), and individual cases of eating disorder, body dysmorphic disorder, post-traumatic stress disorder, and schizophrenia without acute psychotic symptoms. Participants received 8€/h in compensation for their time. All participants gave written informed consent. This study was approved by the ethical committee of University of Münster and was performed in accordance with ethical guidelines specified in the APA Code of Conduct as well as research ethics guidelines in Germany.

**Table 1:** Sample characteristics.

	Clinical	Non-clinical	Group difference
N	25	25	
Mean age (*SD*)	25.4 (7.2)	24.5 (4.2)	*t*(48) = 0.53, *p* = 0.602, *d* = 0.15
Sex			
Women	12	12	χ^2^(1) < 0.01, *p* > 0.999
Men	13	13	
Education			
Low	0	1	χ^2^(2) = 5.20, *p* = 0.074
Middle	4	0	
High	21	24	
Employment status			
In training	18	20	χ^2^(2) = 2.14, *p* = 0.344
Employed	8	5	
Unemployed	1	0	
Symptomatology			
GAD-7	9.2 (4.1)	2.2 (1.6)	*t*(30.9) = 7.97, *p* < 0.001, *d* = 2.25
PHQ-9	11.9 (4.7)	2.7 (1.9)	*t*(31.8) = 9.13, *p* < 0.001, *d* = 2.58

*GAD-7* Generalized Anxiety Disorder Screener, *PHQ-9* Patient Health Questionnaire.

### Symptom levels

Depressive symptoms were assessed using the Patient Health Questionnaire (PHQ-9)^44^, consisting of nine items to assess the symptom severity of depression over the last 2 weeks. Answers are scored on 4-point scales (0 = *not at all*; 3 = *nearly every day*; α = 0.89). Generalized anxiety symptoms were assessed using the Generalized Anxiety Disorder 7 (GAD-7)^45^, comprising seven items about symptoms of generalized anxiety over the last 2 weeks. The GAD-7 is scored on 4-point scales (0 = *not at all*, 3 = *nearly every day*; α = 0.88).

### Diurnal affect diary

Diurnal affect was assessed for a period of seven consecutive days using the Day Reconstruction Method (DRM)^42,46^. For this purpose, participants filled out a pen-and-paper diary, as well as a complemented web survey every evening. The pen-and-paper diary required participants to record all distinct episodes of the respective day (up to 30 per day if needed), to indicate start and end times, and to write down a brief description. They then indicated for each episode how they felt on 11-point scales (0 = *very bad*, 10 = *very good*). Afterwards, participants filled out the web survey, where they reported how they have felt since last filling out the diary, how they felt currently, during the best episode of the day, and during the worst episode of the day, on 11-point scales (0 = *very bad*, 10 = *very good*). For each of these three judgments, they were prompted to substantiate their rating using text boxes. In addition, for the best and worst episodes, they answered a series of items about cognitive engagement with potential comparison standards during the episode, i.e., whether they engaged in social (*In this situation, did you think about other people?*), past temporal (*…your past?*), prospective temporal (*…your future?*), criteria-based (*…how things should be?*), counterfactual (*…how you would feel if certain things had happened or had not happened?*), or dimensional cognitions (*…how you are better in some things than in others?*), on 11-point scales (0 = *not at all*, 10 = *all the time*). For each type of cognition, they also indicated whether it influenced their mood (− 5 = *much worse*, + 5 = *much better*).

To quantify diurnal affect characteristics, we calculated mean affect scores for current, best, and worst affect across the seven days. In addition, we calculated mean affect across all episodes reported in the paper-and-pen diary. We also calculated individual standard deviations and mean squared successive differences (MSSD) as indices of affect variability and temporal instability, respectively^46,47^. 

### Coding

Text responses substantiating the current, best, and worst affect ratings were entered into a coding scheme to classify whether the affect rating was justified by a reference to (1) an external event (yes/no), (2) an internal process like thoughts or feelings (yes/no), and (3) to the respondent’s own activity (yes/no). Next, raters coded (4) the presence of a comparison (present/potentially present/absent); to be coded ‘present’, the response had to mention (a) some comparison standard and (b) relate the standard to the self on some dimension^38^. If one condition (a or b) was unclear (i.e., not explicitly included in the response), comparison was coded as ‘potentially present’. If one condition was not met, comparison was coded ‘absent’. Present and potentially present comparisons were then coded for (5) direction (upward, downward, lateral), (6) comparison type (social, past temporal, criteria-based, counterfactual, prospective temporal, dimensional, contextual), and (7) specificity of the standard (0 = *generic*; 1 = *intermediate*; and 2 = *specific*).

Following consensus agreement between three raters on four transcripts (NM, TM, VM), two raters (VM, TM) coded aspects 4–7 independently of each other for all coding schemes. For aspects 1–3, 30% of the schemes were coded by both raters and the rest by only one rater (TM). Across episodes, mean absolute agreement between raters for aspects 1–3 was 0.871, 0.922, and 0.887, respectively. For aspects 4–6, absolute agreement was 0.801, 0.946, and 0.701, respectively, and 0.614 for the specificity ratings (7). Discrepancies were resolved by a third rater (NM).

### Procedure

Candidates were screened for inclusion and exclusion criteria by telephone (clinical group) or by an online screening (non-clinical group). Eligible participants were then invited to a laboratory session, where they completed the GAD-7 and PHQ-9, a structured clinical interview (clinical group only), and a semi-structured interview about their subjective wellbeing relative to various comparison standards^41^. Afterwards, they received instructions regarding the pen-and-paper diary as well as the daily web surveys, which they then completed every evening for 7 consecutive days. Participants received the links to the daily web surveys via email, each time accompanied by a reminder SMS. Data collection took place in 2018–2019.

### Statistical analysis

The main analyses in the present study focused on engagement with comparison standards (as identified by raters and according to self-report) and the engendered affective impact. Since rater-identified comparisons occurred very infrequently, it was deemed impractical to account for the nested data structure (occurrences within participants and within days) in our statistical approach. Therefore, we summed the number of comparisons per type and direction across all seven days, separately for current, best, and worst episodes, and separately for “present” and “potentially present” comparisons. These frequencies were then subjected to mixed ANOVAs with type of episode (current, best, worst) as within-subjects factor and group (clinical, non-clinical) as between-subjects factor, in addition to either direction (upward, downward), or type of comparison (social vs. past temporal vs. criteria-based vs. counterfactual vs. prospective temporal vs. dimensional), as within-subject factor.

Next, self-reported engagement and affective impact of potential comparisons were analyzed using a linear mixed models (LMM) approach (model type: III sum of squares; test method: Satterthwaite), accounting for the nested data structure, with measurements nested within participants and within days. Main and interaction effects involving group, type of episode (best, worst), and type of standard, were entered as fixed factors, while intercepts of participants and of days were entered as random effects components. Contrasts were explored using Holm corrections. Two separate models addressed engagement with potential comparison standards and affective impact, respectively. Finally, we used a model in which self-reported engagement was entered as a continuous fixed effects variable to predict self-reported affective impact. Interactions in LMM were followed up by contrasting estimated marginal means using Holm correction. When sphericity assumptions or variance homogeneity for ANOVA or t-tests were violated, Greenhouse–Geisser adjusted *p* values are reported along with the respective epsilon and uncorrected degrees of freedom. We report Cohen’s d (in LMM, calculated from contrast *t* and *df*) and η^2^_p_ as effect size estimates.

The sample included completers of a prior interview study^41^ and sample size for the present study was not based on a specific a-priori power analysis. At an alpha level of 0.05, our sample size was adequate for the detection of at least small-to-medium within-between interactions (e.g., *f* ≥ 0.205 for an Episode type × Group interaction) or large differences between two independent samples (*d* ≥ 0.81) with a power (1 − β) = 0.80 (as determined using G*Power V3.1.9). Group differences in sample characteristics were analyzed using IBM SPSS version 28. LMM analyses were conducted JASP^48^. The analyses were not preregistered.

## Results

### Sample characteristics and diurnal affect

Sample characteristics, including mean levels of anxiety and depression, are depicted in Table 1. Number and duration of all recorded DRM episodes (i.e., all episodes that participants could distinguish during the 7-day period), as well as descriptive statistics on the reported valence across episodes, are summarized in Table 2. The reported valence was consistently higher in the non-clinical than in the clinical group for all episode types. This was further confirmed by a 2 (Episode type: best, worst) × 2 (Group: clinical, non-clinical) mixed ANOVA showing a main effect for Episode type, *F*(1,48) = 627.174, *p* < 0.001, η^2^_p_ = 0.929, a main effect for group, *F*(1,48) = 9.059, *p* = 0.004, η^2^_p_ = 0.159, in the absence of an interaction (*p* = 0.750, η^2^_p_ = 0.002).

**Table 2:** Diurnal affect characteristics based on all DRM episodes.

	Clinical	Non-clinical	Group difference
	*M* (*SD*)	*M* (*SD*)	
Episode characteristics			
Total number	55.1 (13.4)	66.7 (24.0)	*t*(38.37) = − 1.948, *p* = 0.059, *d* = − 0.551
Duration (h:mm)	1:57 (0:35)	1:40 (0:27)	*t*(48) = 1.935, *p* = 0.059, *d* = 0.547
Episode valence (0–10)			
All episodes	6.34 (1.12)	7.14 (0.87)	*t*(48) = − 2.809, *p* = 0.007, *d* = − 0.794
Best episode^a,b^	7.92 (0.87)	8.72 (0.78)	*t*(48) = − 3.458, *p* = 0.001, *d* = − 0.978
Worst episode^a,b^	4.46 (1.31)	5.18 (1.04)	*t*(48) = − 2.132, *p* = 0.038, *d* = − 0.603
Current episode^b^	6.07 (1.34)	7.31 (0.89)	*t*(48) = − 3.837, *p* < 0.001, *d* = − 1.085
Since last measure	6.18 (1.24)	7.14 (0.92)	*t*(48) = − 3.120, *p* = 0.003, *d* = − 0.883
Valence variance (SD)	1.39 (0.38)	1.26 (0.33)	*t*(48) = 1.225, *p* = 0.226, *d* = 0.347
Valence stability (MSSD)	3.07 (2.01)	2.92 (1.76)	*t*(48) = 0.270, *p* = 0.788, *d* = 0.076

*MSSD* Mean Squared Successive Differences.

^a^For each of these episodes, participants rated their engagement with comparison standards and the respective affective impact.

^b^For each of these episodes, participants provided a brief explanation of their rating (answers were then coded for the presence of comparisons).

### Rater-coded presence of comparisons

#### Overall frequencies

Recall that we used narrative descriptions of the current, best and worst daily episode to code engagement in comparative behavior during these episodes. Table 3 displays the overall mean comparison numbers across all 21 distinct episodes identified by the raters, using either a strict coding algorithm (i.e. requiring explicit mention of a standard *and* of how the standard relates to oneself) or a more liberal algorithm (i.e., standard and self-relation were implied). As can be seen, only a few comparisons were identified with strict coding (overall *M* = 1.58, *SD* = 2.02), whereas there were more potential comparisons according to liberal coding (overall *M* = 3.90, *SD* = 3.33). Upward and downward comparisons were more frequently used than lateral comparisons. In terms of standard type, comparisons to past temporal and criteria-based standards were most frequently used. Lateral and all other types of comparison were found too infrequently to consider in the following analyses. There were no group differences in comparison numbers, irrespective of comparison type, both when coded strictly (all *p*s > 0.36) and liberally (all *p*s > 0.12).

**Table 3:** Frequency of rater-coded comparisons per direction and standard type.

Comparison type	Group			
	Clinical		Non-clinical	
	*M* (*SD*)	% ≥ 1	*M* (*SD*)	% ≥ 1
Strict coding: *explicitly mentioned* (a) a standard and (b) how it relates to the self				
Any	1.64 (2.12)	56%	1.52 (1.96)	52%
Direction				
Upward	0.84 (1.52)	40%	0.76 (1.16)	44%
Downward	0.60 (1.00)	36%	0.52 (0.82)	36%
Lateral	0.16 (0.47)	12%	0.20 (0.50)	16%
Standard				
Criterion	0.56 (0.96)	32%	0.76 (0.97)	44%
Past	0.64 (1.25)	28%	0.40 (0.76)	28%
Any other type	0.40 (0.76)	24%	0.36 (0.91)	20%
Liberal coding: *mentioned or implied* (a) a standard and (b) how it relates to the self				
Any	4.48 (3.34)	88%	3.32 (3.28)	80%
Direction				
Upward	2.48 (2.52)	80%	1.64 (1.85)	64%
Downward	1.72 (1.57)	68%	1.28 (1.37)	60%
Lateral	0.20 (0.65)	12%	0.32 (0.69)	20%
Standard				
Criterion	1.84 (1.89)	68%	1.44 (1.69)	64%
Past	1.44 (1.50)	60%	1.04 (1.34)	44%
Any other type	1.16 (1.60)	52%	0.84 (1.25)	44%

Comparison numbers did not differ statistically between clinical and non-clinical group.

#### Direction

A 3 (Episode type: current, best, worst) × 2 (Direction: upward, downward) × 2 (Group: clinical, non-clinical) mixed ANOVA on strictly coded comparison frequencies showed an Episode type × Direction interaction, *F*(2,96) = 14.303, ε = 0.834, *p* < 0.001, η^2^_p_ = 0.230, such that downward comparisons were almost never found in the worst episodes (*M* = 0.04, *SE* = 0.03) and occurred more often in the current (*M* = 0.24, *SE* = 0.07; *p*_*Bonferroni*_ = 0.020) and best episodes (*M* = 0.28, *SE* = 0.07; *p*_*Bonferroni*_ = 0.010). Meanwhile, upward comparisons were found more often in the worst episodes (*M* = 0.52, *SE* = 0.12), compared to current (*M* = 0.22, *SE* = 0.07; *p*_*Bonferroni*_ = 0.014), and best episodes (*M* = 0.06, *SE* = 0.03; *p*_*Bonferroni*_ < 0.001), where they were close to never present. The ANOVA did not show any effect involving Group (all ps > 0.164).

The same 3 × 2 × 2 ANOVA on liberally coded comparison frequencies yielded highly similar results with larger effect sizes, i.e., an Episode type × Direction interaction, *F*(2,96) = 39.614, *p* < 0.001, η^2^_p_ = 0.452. Again, downward comparisons rarely occurred in the worst episodes (*M* = 0.10, *SE* = 0.04) and more often in the current (*M* = 0.50, *SE* = 0.10; *p*_*Bonferroni*_ < 0.001) and best episodes (*M* = 0.90, *SE* = 0.14; *p*_*Bonferroni*_ < 0.001). Upward comparisons were more frequent in the worst episodes (*M* = 1.30, *SE* = 0.20), and less frequent in the current (*M* = 0.60, *SE* = 0.12; *p*_*Bonferroni*_ < 0.001) and best episodes (*M* = 0.14, *SE* = 0.05; *p*_*Bonferroni*_ < 0.001).

#### Standard type

A 3 (Episode type: current, best, worst) × 2 (Standard: past temporal, criterion) × 2 (Group: clinical, non-clinical) mixed ANOVA on strictly coded comparison frequencies did not reveal any three-way interaction (*p* = 0.946), two-way interaction (all *p*s > 0.196), or main effect (all *p*s > 0.368). However, the same ANOVA on liberally coded frequencies yielded an Episode × Standard interaction, *F*(2,96) = 9.289, *p* < 0.001, η^2^_p_ = 0.162 in the absence of any Group effects (all ps > 0.271). The past temporal standard was relatively infrequent in the worst episode (*M* = 0.22, *SE* = 0.07) and found more often in the best episodes (*M* = 0.58, *SE* = 0.11; *p*_*Bonferroni*_ = 0.011), with no difference to the current episodes (*M* = 0.44, *SE* = 0.10; *p*_*Bonferroni*_ = 0.124). Conversely, criteria-based comparisons were relatively infrequent in the best episodes (*M* = 0.24, *SE* = 0.07) and were more often found in the current episodes (*M* = 0.58, *SE* = 0.13; *p*_*Bonferroni*_ = 0.038), and most frequently in the worst episodes (*M* = 0.82, *SE* = 0.17; *p*_*Bonferroni*_ = 0.010).

#### Concreteness

We compared mean concreteness ratings (0–2) across all coded comparisons between the clinical and the non-clinical groups, including only participants with at least one comparison. No significant differences emerged both for strictly coded comparisons (clinical: *n* = 14, *M* = 1.08, *SD* = 0.63; non-clinical: *n* = 13, *M* = 1.24, *SD* = 0.59; *d* = − 0.266, *p* = 0.496) and for liberally coded comparisons (clinical: *n* = 22, *M* = 0.77, *SD* = 0.52; non-clinical: *n* = 20, *M* = 1.02, *SD* = 0.49; *d* = − 0.504, *p* = 0.111), although the clinical group had descriptively lower concreteness ratings on average.

### Self-reported engagement with potential comparison standards

**Figure 1:**
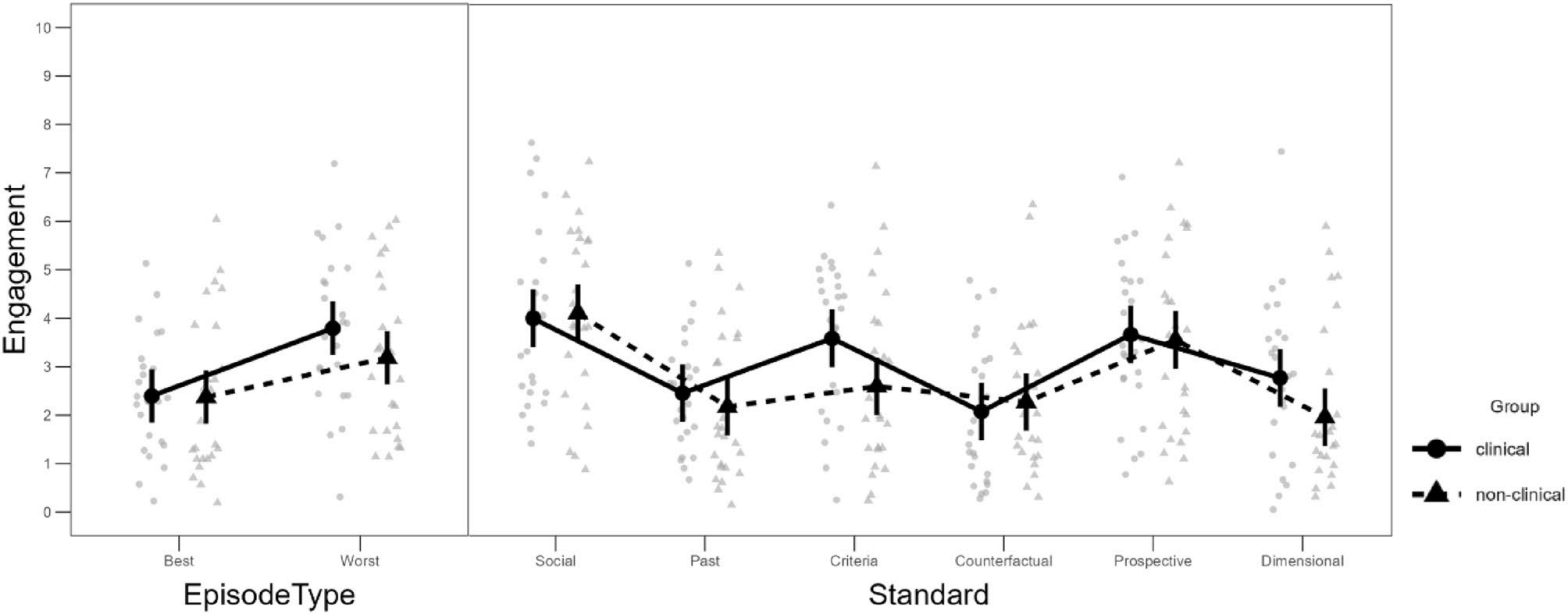
Estimated mean frequencies of engagement per group in the best/worst daily episode (left panel) and with each standard type (right panel). The background data represent estimates per participant. For reasons of visibility, these estimates are aggregated across all days. Error bars denote 95% CI of the estimated marginal means.

The full models of all LMM analyses are provided via the Open Science Framework and can be inspected using the following link: https://osf.io/uwhbp/. For self-reported engagement frequencies, the LMM analysis revealed main effects of Episode type (i.e., more engagement during worst compared to best episodes; *d* = 0.43), *F*(1,4086.1) = 188.75, *p* < 0.001, and Standard type (social, past temporal, criteria-based, counterfactual, prospective temporal, dimensional), *F*(5,4086.1) = 62.26, *p* < 0.001, both of which were qualified by interactions with Group (clinical, non-clinical); Episode type × Group: *F* = 13.26, *p* < 0.001, Standard type × Group *F* = 6.14, *p* < 0.001, see Fig. 1. In addition, there was a significant Episode type × Standard type interaction, *F*(5,4086.1) = 28.67, *p* < 0.001. There was no three-way interaction (*p* = 0.118).

Contrasts confirmed the Episode type × Group interaction (*p*_*Holm*_ < 0.001, *d* = 0.11), which was due to a relatively larger effect of Episode in the clinical group, but we identified no significant simple effects of Group (*p*_*Holm*_ ≥ 0.247, *d* < 0.43). Regarding the Standard type × Group interaction, there were also no significant simple effects for Group per Standard type after correcting for multiple comparisons, whereby the largest descriptive group concerned engagement with criteria-based standards, (*p*_*Holm*_ = 0.137, *d* = 0.55; uncorrected *p* = 0.023). An additional contrast exploring the Standard type × Group interaction indicated that the interaction can be partly attributed to differences between criteria-based and counterfactual standards. That is, the clinical group engaged relatively more in criteria-based than in counterfactual comparisons, which difference was smaller in the non-clinical group (*p*_*Holm*_ < 0.001, *d* = 0.13).

Finally, we explored the Episode type × Standard type interaction with contrasts (across groups), revealing that engagement was generally larger during worst episodes for all standards, *p*_*Holm*_ < 0.001, *d* > 0.10, with the exception of the social standard (*p*_*Holm*_ = 0.159, *d* = 0.04). The largest simple effect of Episode type was present for the criteria-based standard, (*p*_*Holm*_ < 0.001, *d* = 0.46).

### Self-reported affective impact of engagement with comparison standards

**Figure 2:**
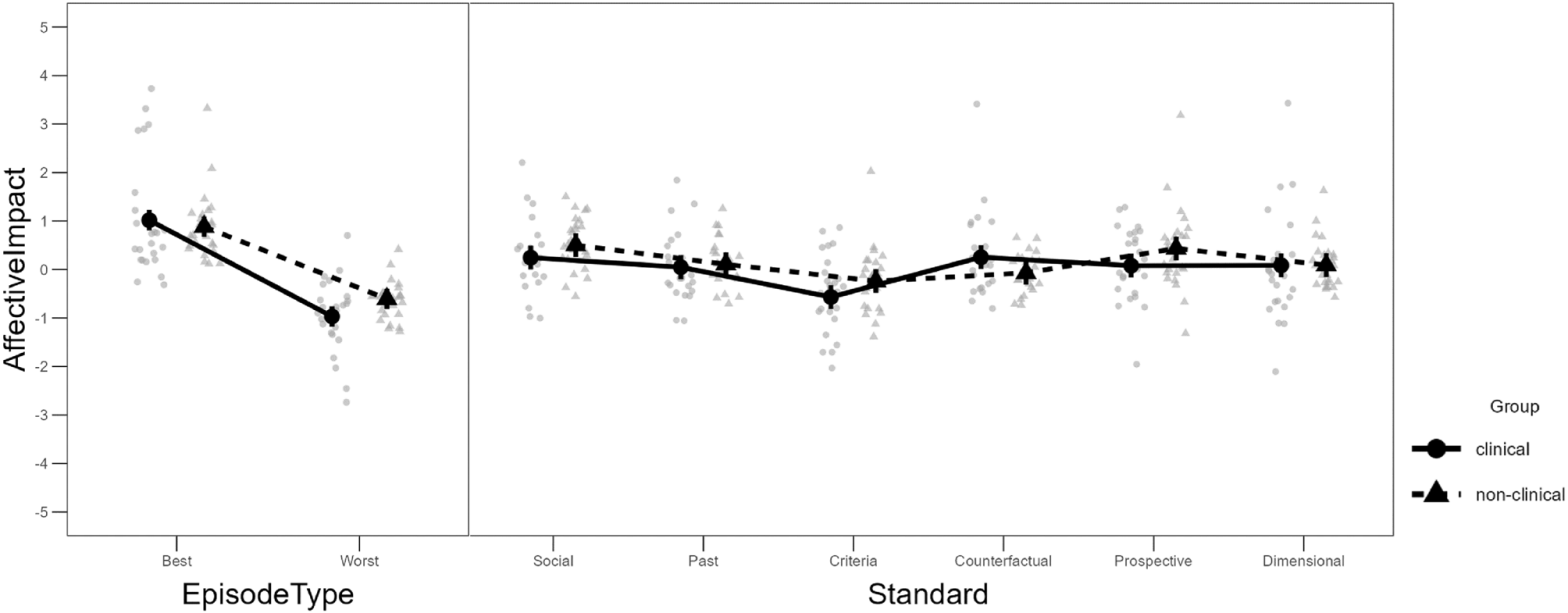
Estimated mean affective impact per group in the best/worst daily episodes (left panel) and with each standard type (right panel). The background data represent estimates per participant. For reasons of visibility, these estimates are aggregated across all days. Error bars denote 95% CI of the estimated marginal means.

LMM for self-reported affective impact following engagement with the potential comparison standards again revealed main effects of Episode type (best, worst; direct contrast *d* = 1.18), *F*(1,4086.1) = 1421.76, *p* < 0.001, and Standard type, *F*(5,4086.1) = 22.17, *p* < 0.001. Again, both effects were qualified by interactions with Group (clinical, non-clinical); Episode type × Group: *F* = 29.21, *p* < 0.001, Standard type × Group *F* = 5.18, *p* < 0.001, see Fig. 2. Further, there was a significant Episode type × Standard type interaction, *F*(5,4086.1) = 28.36, *p* < 0.001, in the absence of a three-way interaction (*p* = 0.458).

Contrasts addressing the Episode type × Group interaction (*p*_*Holm*_ < 0.001, *d* = 0.17) revealed that during worst episodes, the clinical group reported a more negative affective impact than the non-clinical group, *p*_*Holm*_ = 0.036, *d* = − 0.64. No such difference was found in best episodes, *p*_*Holm*_ = 0.366, *d* = 0.24. With respect to the Group × Standard type interaction, contrasts did not reveal any simple effect per Standard type, *p*_*Holm*_ > 0.272. Rather, the interaction appeared to stem from a difference in affective impact between criteria-based and counterfactual standards, which was larger in the clinical than in the non-clinical group, *p*_*Holm*_ < 0.001, *d* = 0.13. That is, the clinical group had a relatively more positive impact in response to counterfactual standards, or a relatively more negative impact to criteria-based standards, as compared with the non-clinical group.

The Episode type × Standard type interaction (across groups) indicated that affective impact was generally lower during worst episodes than during best episodes (*p*_*Holm*_ < 0.001, *d* > 0.28), whereby the difference was the largest for the social standard (*d* = 0.75). Within best episodes, the social standard also had a more positive affective impact compared to all other standards (*p*_*Holm*_ < 0.001, *d* = 0.34). Within worst episodes, criteria-based standards had a more negative affective impact compared to all other standards (*p*_*Holm*_ < 0.001, *d* = − 0.28).

### Conditional associations between engagement with standards and affective impact

The LMM analysis of self-reported affective impact with engagement as an additional continuous fixed effects variable revealed a Group × Episode type × Engagement interaction, *F*(1,4086.1) = 1421.76, *p* < 0.001. Furthermore, there was an Episode Type × Standard type × Engagement interaction, *F*(5,4068.8) = 13.95, *p* < 0.001. Contrasts of slopes revealed that during worst episodes, the clinical group displayed a more negative association between engagement and affective impact (estimated slope = − 0.354, 95% CI − 0.381; − 0.327) than the non-clinical group (estimated slope = − 0.265, 95% CI − 0.294; − 0.236), *p*_*Holm*_ < 0.001, *d* = − 0.96. The respective slopes in the best episodes were small and did not differ significantly (*p*_*Holm*_ = 0.891), even though they tended to be negative in the clinical (estimated slope = − 0.103, 95% CI − 0.135; − 0.072) and positive in the non-clinical group (estimated slope = 0.107, 95% CI = 0.074; 0.139). Meanwhile, the Episode Type × Standard type × Engagement interaction was most likely attributable to the fact that engagement was generally associated with a more negative impact in worst episodes than in best episodes across all standard types, *p*_*Holm*_ < 0.001, *d* < − 0.19, which effect was most pronounced for the social standard, *d* = − 0.55.

### Rater-coded type of explanation

To explore effects involving the type of explanation provided by participants for each episode, we ran a 3 (Episode type: current, best, worst) × 3 (Explanation type: external event, internal process, activity) × 2 (Group: clinical, non-clinical) mixed ANOVA. This indicated an Episode by Explanation interaction, *F*(4,192) = 26.798, *p* < 0.001, η^2^_p_ = 0.358, in the absence of effects involving Group (all *p*s > 0.190). External events were least commonly reported in the worst episode of the day (M = 40.7%, SE = 3.0%), and more often in the best (M = 53.5%, SE = 3.3%) and current episodes (M = 60.6%, SE = 3.7%), *p*s_*Bonferroni*_ ≤ 0.002. By contrast, internal processes were reported most frequently in the worst episode (M = 83.3%, SE = 3.0%), and more so than in the best (M = 65.2%, SE = 3.7%) and the current episodes (M = 72.8%, SE = 3.7%), *p*s_*Bonferroni*_ ≤ 0.002. Finally, respondents referred to their own activity most commonly in the best episode (M = 83.0%, SE = 2.7%), and less often in the worst (M = 51.9%, SE = 3.6%) and in the current episodes (M = 56.6%, SE = 3.6%), *p*s_*Bonferroni*_ < 0.001.

## Discussion

We investigated whether judgments of subjective wellbeing are informed by different types of comparison, with standards varying within-person (i.e., best vs. worst daily episodes), and between clinical vs. non-clinical participants. Overall, best daily episodes were characterized by positive affective impact across all potential comparison standards (social, past temporal, criteria-based, counterfactual, prospective temporal, dimensional), and we most frequently identified downward and past-temporal comparisons. In contrast, worst episodes were characterized by negative affective impact across all potential standards, whereby we most often identified upward and criteria-based comparisons. Compared to non-clinical participants, the clinical group had consistently lower affect levels, engaged more often with criteria-based relative to other standards (e.g., counterfactual), and generally engaged more strongly with any type of comparison standard during worst compared to best episodes. Critically, engagement with potential comparison standards during worst episodes was also more strongly linked to negative affective impact in the clinical group. These findings suggest that comparative thinking underlies judgments of affect and wellbeing, whereby certain standards (e.g., criteria-based) may be particularly involved in negative affect and poor mental health.

Our data supported the expectation that episodes of low wellbeing (i.e., worst daily episodes) are characterized generally by more upward than downward comparisons, relative to episodes with high wellbeing (i.e., best daily episodes). This aligns with the typical finding in cross-sectional, experimental and diary studies that upward comparison results in worse affect while downward comparison is followed by better affect^32–36^, an effect that has been robustly found in social comparison studies^17^. Moreover, factor analytical studies indicate that for the majority of standards (e.g., social, past-temporal, criteria-based), upward comparison is generally motivationally aversive, whereas downward comparison is appetitive^22,26,31^. Thus, the present study replicates and extends these findings by showing that (a) people tend to invoke upward comparison to explain their wellbeing during worst daily episodes, whereas they virtually never invoke downward comparisons, (b) the opposite pattern was found for best daily episodes, (c) in worst and best episodes, people more likely mention certain criteria or their past, respectively, (d) engagement with all comparison standards has negative affective impact during worst and positive impact during best episodes, and (e) that these patterns can be found in both in clinical and non-clinical individuals.

Meanwhile, our analyses involving between-subjects effects may be somewhat surprising as they lend only limited support to the idea that individuals suffering from a mental disorder generally engage more frequently in unfavorable comparisons^20,37^. That is, amid consistently lower levels of wellbeing, we found no differences between groups regarding the number of comparisons they used to substantiate their wellbeing score during the different episode types. Instead, the clinical group reported generally more pronounced engagement with potential comparison standards in worst compared to best episodes, accompanied by more negative impact. Additionally, more subtle group differences indicate that the clinical group engages relatively more often in thoughts about criteria-based standards having a negative affective impact, than in counterfactual comparisons having a more positive impact. Moreover, there were no differences in the concreteness of the comparison standards, or type of explanation provided. This may seem surprising in light of our prior study using a wellbeing interview^41^, in which we found relatively large differences between the clinical and non-clinical groups, such as generally more comparisons in the clinical group (*d* = 1.46), and more upward comparisons in particular (*d* = 1.01). One explanation for the current findings is that these group differences do not translate to strong differences moment-to-moment comparative processes between clinical and non-clinical populations. This makes sense insofar as a one-off assessment concerning comparisons in relation to wellbeing invites more global and long-term considerations about one’s life. In contrast, the focus of the present study—momentary wellbeing per episode—may invite thoughts about closer frames of reference (e.g., spatially, temporally) that may be relatively similar in clinical and non-clinical groups. Indeed, this aligns with the finding that both groups similarly refer to their own activities, external events, or internal thought processes, when explaining their current wellbeing without any significant differences regarding the frequency of comparisons. Importantly, lower judgments of wellbeing in the clinical group relative to the non-clinical group might also be the result of some individual comparisons exerting a stronger impact in the clinical group than the non-clinical group. Yet, our study was not designed to assess the impact of single comparisons on wellbeing judgments and hence this remains an open question for future research.

### Limitations and considerations for future research

It is important to note that prior to completing this diary study, our participants had enrolled in an interview study addressing comparative thinking^41^. This may have led some participants to be more aware of their comparative thinking and behaviours than usual, despite our attempts to minimize demand and expectancy biases by (a) relying on open-ended rather than closed questions and (b) omitting explicit reference to comparisons when asking about engagement with the different standards. However, the finding that our open-ended questions per episode yielded a relatively low number of comparisons, even with liberal coding, suggests negligible demand characteristics. Instead, comparative thinking may often occur without people being aware of it. Hence, a different limitation is that our open-ended questions may have been relatively insensitive to capture comparative thinking. Indeed, a 5-min long interview appears to yield much higher numbers of comparison by allowing more fine-grained differentiation of comparison types, in particular when respondents are directly prompted about different standards^41^. Relatedly, we asked participants about engagement with potential comparison standards (“have you thought about other persons?”), but this may not always correspond to engagement in comparison, which additionally requires relating the standard to the self^49^. A more general potential limitation is that we did not control for various individual and sociodemographic factors that may influence reporting of comparative behaviour (e.g., intelligence, socioeconomic status, important biographical events), which need to be considered in future studies. Finally, our sample size was rather small to identify overall between-group differences, implying that statistical power may have been adequate only for between-within interactions and for larger effect sizes. Thus, absence of effects should not be inferred from our data—in particular with regards to smaller effects that may have gone undetected—and replication with larger samples is clearly warranted.

A noteworthy observation was that participants in the clinical group tended to report fewer daily and longer episodes than the non-clinical group (see Table 2). Although this difference was not significant, such a pattern could be explicable by a combination of lower levels of activity in patients along with possible distortions in memory recollection, such as a difficulty to retrieve specific episodes^50^. Therefore, future studies addressing comparative thinking in everyday life should take the potential roles of activity levels and memory processes into account.

## Conclusions

Our study shows that fluctuating judgments of affect and wellbeing are associated with comparative thinking, such that upward and downward comparisons, criteria-based and past-temporal comparisons, and references to internal thoughts and own activities tend to characterize worse and best daily episodes, respectively. These diurnal patterns of changing comparison standards appear to be similar in clinical and non-clinical groups to a large extend, whereby the clinical group displayed a more pronounced engagement with aversive standards. Moreover, certain standards (e.g., criteria-based) may play a particularly important role in negative affect and poor mental health, warranting further careful research. Future studies may extend these findings by investigating more nuanced differences in comparative thinking, e.g., in specific patient subgroups or during certain types of daily episode.

## Data Availability

The anonymized datasets obtained and analyzed for the current study are available in the Open Science Framework repository, using the following link: https://osf.io/uwhbp/.
